# The Role of Galectin-3 in Predicting Congenital Heart Disease Outcome: A Review of the Literature

**DOI:** 10.3390/ijms241310511

**Published:** 2023-06-22

**Authors:** Amalia Făgărășan, Maria Săsăran, Liliana Gozar, Andrei Crauciuc, Claudia Bănescu

**Affiliations:** 1 Department of Pediatrics III, Faculty of Medicine, George Emil Palade University of Medicine, Pharmacy, Sciences and Technology of Târgu Mures, Gheorghe Marinescu Street no 38, 540136 Târgu Mures, Romania; amalia_fagarasan@yahoo.com (A.F.); lili_gozar@yahoo.com (L.G.); 2 Department of Pediatrics III, Faculty of Medicine in English, George Emil Palade University of Medicine, Pharmacy, Sciences and Technology of Târgu Mures, Gheorghe Marinescu Street no 38, 540136 Târgu Mures, Romania; 3 Department of Medical Genetics, George Emil Palade University of Medicine, Pharmacy, Sciences and Technology of Târgu Mures, Gheorghe Marinescu Street no 38, 540136 Târgu Mures, Romania; andrei.crauciuc@gmail.com; 4 Genetics Department, Center for Advanced Medical and Pharmaceutical Research, George Emil Palade University of Medicine, Pharmacy, Science, and Technology of Târgu Mureș, Gheorghe Marinescu Street no 38, 540136 Târgu Mures, Romania; claudia.banescu@gmail.com

**Keywords:** galectin-3, congenital heart disease, plasma biomarker, heart failure, ventricular dysfunction

## Abstract

Galectin-3 (Gal-3) is a novel pro-fibrotic biomarker that can predict both right and left cardiac dysfunction caused by various cardiovascular conditions. Its expression seems to be progressively altered with evolving cardiac remodeling processes, even before the onset of heart failure. Hence, Gal-3 has been found to be an individual predictor of acute and chronic heart failure or to serve as part of an integrated biomarker panel that can foresee adverse cardiac outcomes. In congenital heart disease (CHD), Gal-3 correlates with cardiac mortality and complications in both children and adults and is proposed as a therapeutic target in order to reverse the activation of pro-fibrosis pathways that lead to heart failure. Positive associations between serum Gal-3 levels, post-operatory hospitalization rates, complications and ventricular dysfunction have also been reported within studies conducted on patients with CHD who underwent corrective surgery. Thus, this review tried to address the potential utility of Gal-3 in patients with CHD and particularly in those who undergo corrective surgery. The heterogeneity of the literature data and the lack of validation of the results obtained by the current studies on larger cohorts cannot be neglected, though. Further longitudinal research is required to establish how Gal-3 can relate to long-term outcomes in pediatric CHD.

## 1. Introduction

Galectin-3 (Gal-3) represents a β-galactosidase-binding lectin that can mediate cell-to-extracellular matrix (ECM) communication through interactions with ligands such as laminin, integrin and collagen [1]. Although mainly found within the cellular cytoplasmatic compartment, Gal-3 has been identified both within the nucleus and the mitochondria and can be easily externalized through specific exosomes by an alternative secretory pathway [2,3,4]. Its proven involvement in cellular pre-messenger RNA (mRNA) splicing within the nucleus implies a direct correlation with cellular proliferation processes, which has been strengthened by the discovery of its antiapoptotic effects exerted through the inhibition of cytochrome c release and mitochondrial protection [3,5]. As Gal-3 has the ability to bind to both the cell surface and the ECM and to interact with cellular components, multiple experimental studies have proposed this galactosidase as a mediator of the pathways involved in cellular apoptosis, angiogenesis, cell adhesion, migration and inflammation [6]. A multifunctional protein with both intracellular and extracellular roles, Gal-3 is expressed by several types of cells, such as epithelial cells, myeloid cells and immune cells (neutrophils, B- and T-cell lymphocytes, macrophages and natural killer cells) [7]. Moreover, Gal-3 has been identified within the conjunctive, epithelial tissue, respiratory tract, digestive tract, urinary tract and hepatic and heart tissue [8]. Given its complex interaction with cytosolic proteins, as well as its ubiquitous tissular expression, Gal-3 has been proven to play a role in the development of a variety of disorders, including renal disease, cardiovascular disorders, viral infection, autoimmune diseases, neurodegenerative disorders and malignancies [9,10,11,12,13,14,15,16].

**Figure 1:**
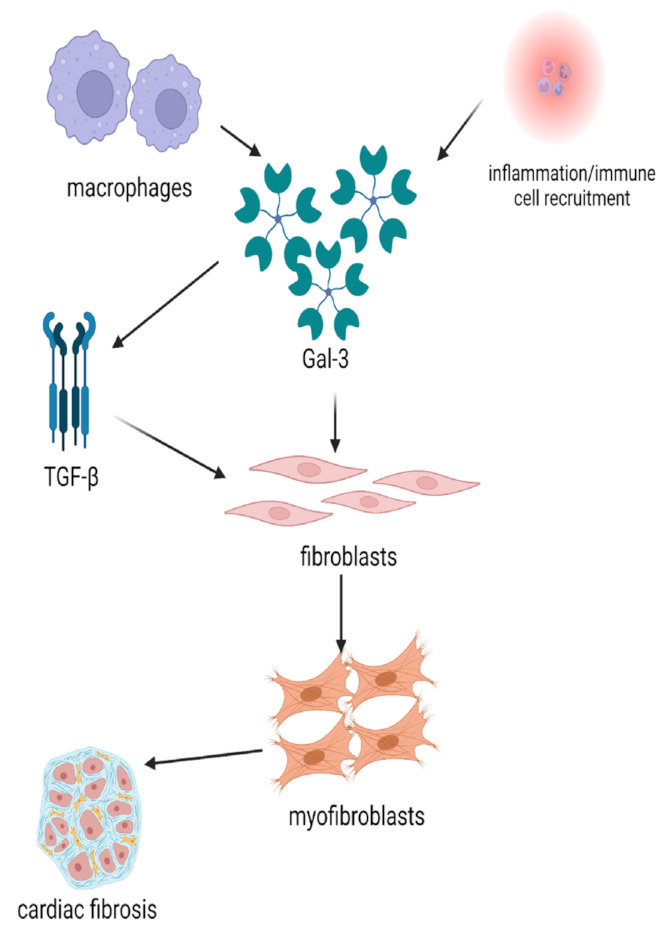
Gal-3 involvement in the pathogenesis of cardiac fibrosis (created with BioRender.com). Legend: Gal-3—galectin-3; TGF-β—transforming growth factor-β.

Gal-3 has been regarded as a key effector in the promotion of fibrosis after the initial disproportionate inflammation-driven processes, which will enhance the transformation of fibroblasts into myofibroblasts and, therefore, the expansion of the ECM, a mandatory stage in fibrotic tissue genesis [6]. This mechanism is mediated by transforming growth factor-beta (TGF-β) and macrophages, which both promote myofibroblast differentiation [17,18]. It has been known for a long time that Gal-3 is overexpressed within macrophages, as proven by early experimental studies [19]. Moreover, Gal-3 stimulates TGF-β activity and has been proposed as a therapeutic target in the mediation of idiopathic pulmonary fibrosis [20]. Excessive macrophage aggregation and inducement of the TGF-β1/α-smooth muscle actin (SMA)/collagen I (Col I) profibrotic pathway represents essential physiopathological processes leading to atrial fibrosis, according to experimental studies conducted on rats [21,22,23]. The involvement of Gal-3 in the pathogenesis of cardiac fibrosis is briefly illustrated in Figure 1.

Cardiac fibrosis represents a hot topic for the in vitro modulation of Gal-3 expression. Its proven involvement in the renin–angiotensin–aldosterone axis led to the proposal of its inhibition as a therapeutic tool for the prevention of myocardial hypertrophy and interstitial fibrosis [24]. For example, spironolactone administration reduces the Gal-3 concentrations in patients with reduced ejection fractions, which shows a potential beneficial therapeutic outcome of the inhibition of the renin–angiotensin–aldosterone axis for the prevention of myocardial fibrosis and remodeling processes [25]. However, there is a dispute surrounding the cardiac specificity of Gal-3 due to its persistent elevation in patients before and after heart transplantation. There is also a lack of correlation between the circulating levels and Gal-3 expression within myocardial tissue and in post-transplant interstitial fibrosis [26].

**Figure 2:**
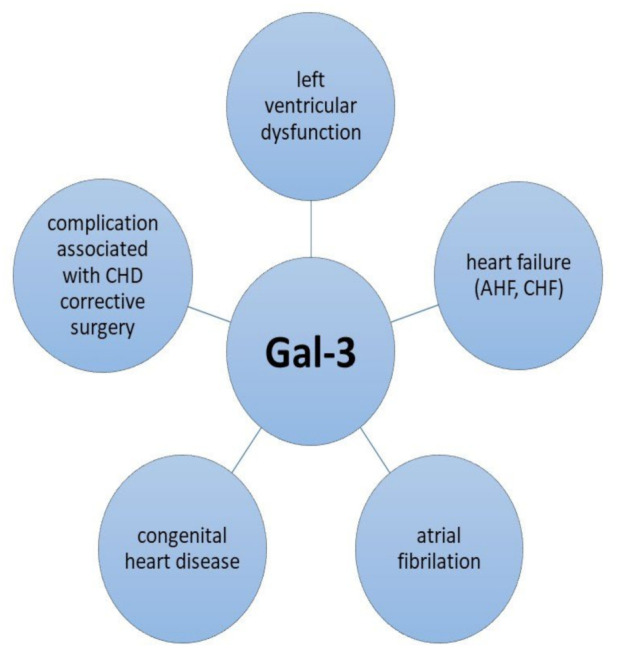
Gal-3 and its associations with various cardiovascular disorders. Legend: AHF—acute heart failure; CHF—chronic heart failure; CHD—congenital heart disease.

Starting from evidence based on the active role of Gal-3 in the modulation of cardiac fibrosis, multiple research articles have proposed this molecule as a biomarker for various cardiovascular diseases, including cardiac insufficiency, atrial fibrillation and congenital cardiac malformations, or as a predictor of long-term outcomes after cardiac resynchronization therapy [27,28,29,30,31]. As most chronic heart disease conditions will eventually lead to the development of chronic heart failure (CHF), the role of Gal-3 as a prognostic factor and mortality predictor in patients diagnosed with severe myocardial disfunction has been thoroughly investigated [32,33,34]. Figure 2 provides a synopsis of the associations between Gal-3 and various cardiovascular disorders.

The present review aims to assess the role of Gal-3 in predicting myocardial dysfunction, as well as specific complications related to cardiac remodeling processes in congenital cardiac malformation. Starting from extensive research that asserted Gal-3 as a marker of ventricular maladaptation and its utility in the follow-up of patients with a diagnosis of heart failure of different etiologies, this review will try to highlight the potential utility of this pro-fibrotic marker in predicting the early onset of heart failure signs and post-operatory complications of corrective surgery in patients with congenital heart disease (CHD).

## 2. Materials and Methods

After an initial screening of the literature data for Gal-3-based physiopathological mechanisms involved in cardiac remodeling and heart failure, which constitute the grounds upon which Gal-3 has emerged as a biomarker in CHD, our focus was directed toward the role of Gal-3 in CHD and its outcome. The PubMed, Web of Science and Scopus databases were searched for articles published in the English language that assessed the role of Gal-3 in CHD, it complications and corrective cardiac surgery outcomes. The search terms were “Gal-3” AND “congenital heart disease” OR “congenital cardiac malformation” OR “congenital heart defect”. We included adult and pediatric population-based studies that adhered to our aforementioned objectives. Experimental, animal-based studies and studies published in a non-English language were excluded.

## 3. Gal-3 and Cardiac Remodeling

### 3.1. Gal-3 and Cardiac Fibrosis

Gal-3 was initially thought to be a marker of progressive cardiac remodeling associated with left ventricular dysfunction and positively correlated with the rate of adverse cardiovascular events [35]. However, Gal-3 expression within the myocardium seems to be altered before the onset of heart failure, as suggested by an experimental study conducted on pericardial Gal-3-infused rats, which exhibited significant collagen production triggered by cardiac fibroblasts [36]. The in vitro pharmacological inhibition of Gal-3 prevented the development of heart failure by hampering cardiac fibrosis and left ventricular dysfunction. Still, left ventricular hypertrophy continued to develop as a result of the intervention used to trigger cardiac remodeling, even with Gal-3 inhibition [37]. Thus, Gal-3 seems to mainly interfere with collagen production. An in vivo example is represented by the onset of cardiac remodeling caused by volume overload in the setting of arterial hypertension, which can already lead to a significant elevation in Gal-3 [38].

### 3.2. Gal-3 and Left Ventricular Remodeling

The relationship between Gal-3 and left ventricular remodeling is mainly reflected through a low LVEF. Elevated Gal-3, together with increased values of N-terminal pro-B-type natriuretic peptide (NT-proBNP) and C-reactive protein (CRP) were predictive of a very diminished left ventricular ejection fraction (LVEF), with values even lower than 35%, in patients with newly diagnosed dilated cardiomyopathy, according to Rieth et al. [39]. This association between Gal-3 and diminished LVEF was further strengthened by changes seen on cardiac magnetic resonance imaging in subjects who suffered from myocardial infarction [40,41]. These changes often became obvious 30 days post-myocardial infarction, in correlation with diastolic dysfunction progression [42]. It is thought worth mentioning that impaired parietal kinetics has also been significantly associated with mid-range ejection fraction in heart failure patients [43]. However, other studies, such as the one conducted by de Boer et al., showed that the prognostic value of Gal-3 stands out only in those patients with HF and preserved left ventricular ejection fraction (LVEF) [44]. One meta-analysis claimed that Gal-3 can be used as a predictor for new-onset HF with preserved LVEF, which also correlates with left ventricular diastolic dysfunction [45]. A recent review article confirmed these findings and proposed Gal-3 as a marker that can guide the therapeutic approach in HF with preserved LVEF [46]. Still, due to the paucity of studies that have assessed the Gal-3 levels in subjects with HF and preserved LVEF, another recent meta-analysis addressed the need for future research in this particular clinical setting [47].

### 3.3. Gal-3 and Atrial Remodeling

Gal-3 poses pro-fibrotic effects upon the atrial interstitium, promoting arrhythmogenesis through CD98 (its membrane surface receptor) signaling [48]. As fibrosis is the main precipitant factor for the development of atrial fibrillation (AF), the biomarker role of Gal-3 in AF has been proposed [49]. Since AF usually occurs in the setting of cardiac structural remodeling, namely atrial dilation and cardiomyocyte replacement by fibrotic tissue, research testifying a correlation between its higher incidence in the general population and elevation in Gal-3 levels was foreseeable [50,51]. Moreover, the AF severity and persistence were related to higher Gal-3 values by Ho et al. but without taking into account additional risk factors [51]. Thus, Gal-3 has been proposed as a therapeutic guidance adjuvant that can predict the need for renin–angiotensin–aldosterone system inhibitor therapy, as well as a potential therapeutic target, with the help of β-adrenoceptor antagonists. As a result, conversion to a sinus rhythm can be more easily achieved through the pharmacological modulation of Gal-3 [52,53,54]. Gal-3 might also be useful in the prediction of post-operative AF. An elevation in Gal-3 levels can predict AF occurring after coronary artery bypass surgery, according to Erdem et al. In their study, patients who maintained a sinus rhythm post-operatively had significantly lower Gal-3 levels, but these values were not compared with the ones of healthy controls [55]. Other studies have also related the augmentation of Gal-3 with the arousal of AF after cardiac surgery, but a recent review of available evidence concluded that there was insufficient data available due to contradictory results reported and the limited study samples on which the research on the matter was conducted [56,57,58].

### 3.4. Gal-3 and Cardiac Remodeling in Relation to Impaired Glucose Metabolism

The relationship between Gal-3 and cardiac remodeling in altered glucose tolerance or diabetes mellitus patients is still being debated. Gal-3 might represent a predictor of early cardiac remodeling in patients with diabetes mellitus but only in junction with other cardiac-specific biomarkers [59]. Moreover, elevation of the Gal-3 levels was reported in both pre-diabetic and diabetic patients within a case–control study without any relationship to the cardiac function or adverse events [60]. In spite of existing proof regarding the augmentation of Gal-3 in patients with heart failure and impaired glucose metabolism, the reduction of the left ventricular contractile reserve among patients with heart failure and diabetes mellitus did not independently alter the plasmatic Gal-3 levels.

## 4. Gal-3, Heart Failure and Its Associated Complications

Gal-3 has been established as a marker in multiple cardiovascular diseases. Its connection to heart failure has been widely investigated and reviewed in multiple publications, which presented Gal-3 as an useful tool for risk stratification and prognosis evaluation in both acute and chronic heart failure [32,61,62]. This section provides a brief presentation of the role of Gal-3 in acute, incidental and chronic heart failure.

### 4.1. Gal-3 and Acute Heart Failure

Van Kimmenade et al. were the first to evaluate the utility of novel serum markers in the diagnosis of acute heart failure (AHF). The authors concluded that plasma NT-proBNP represents the best diagnostic marker, with a superiority over both apelin and Gal-3, but suggested that the combination between NT-proBNP and Gal-3 might be the best mortality predictor [63]. In a similar fashion, a review performed on pediatric studies that enrolled patients diagnosed with heart failure concluded that none of the currently available serum biomarkers of myocardial fibrosis perform as well as NT-proBNP in predicting ventricular dysfunction [64]. Gal-3 was afterwards regarded by the randomized SHOCK-COOL trial as a stable biomarker that correlates well with a 30-day mortality of different causes in patients with AHF and which is not influenced by age, sex and body mass index [65]. It is therefore unsurprising that Gal-3 has been proposed as a diagnostic biomarker and potential therapeutic target in AHF [66]. Still, Gal-3 values seem to be dependent upon renal function, with one large-scale study suggesting that the serum levels of Gal-3 need to be adjusted according to the glomerular filtration rate [67]. As a matter of fact, recent research in the field recommends the use of a multi-marker panel for evaluation of the AHF prognosis, which includes Gal-3 and creatinine, as well as growth differentiation factor 15 (GDF-15), NT-proBNP, suppression of tumorigenicity 2 (ST2) and troponin I [68,69]. Wang et al. even sustained that a combined biomarker panel consisting of Gal-3, NT-proBNP and ST2 might be indicative of systemic fibrotic processes evolution, not only of those localized within myocardial tissue [69].

### 4.2. Gal-3 and Incident Heart Failure

Gal-3 concentrations correlate with incident heart failure risk, as claimed by a meta-analysis conducted on nine studies [70]. Gal-3 elevation enhances the risk of incident heart failure and is associated with higher overall mortality rates and increases in the left ventricular mass, according to Ho et al. [71]. The ARIC study also identified a connection between Gal-3 elevation and incident heart failure but emphasized that Gal-3 values tend to be higher in the obese, especially among females and Caucasian populations [72]. A study conducted on a large population-based cohort (FINIRISK 1997 cohort) initially established that Gal-3 counts as a predictor of three major endpoints—namely, all-cause mortality, cardiovascular death and incident heart failure—after adjusting for the parameters of the Framingham risk score. However, when additionally adjusting for NT-proBNP, the Gal-3 value was not related to an increase in heart failure risk anymore [73]. Still, another meta-analysis of 18 studies concluded that high Gal-3 levels are correlated with incidental heart failure, all-cause mortality and cardiovascular mortality in the general population [74].

### 4.3. Gal-3 and Chronic Heart Failure

In the case of CHF, Gal-3 also acts as an important predicting factor independent of the severity of HF assessed through the NT-proBNP levels [75]. A cut-off level of 11.5 ng/mL has been proven to be predictive of CHF exacerbations requiring hospitalization, according to Baran et al., who also identified a correlation between Gal-3 and vascular stiffness within their study group [76]. Other research proposed a cut-off value of 8 ng/mL as a risk factor for CHF development. This case–control study also reported an increase in the Gal-3 levels with age among the healthy subjects [77]. The symptom severity, morbidity and hospitalization rates in patients with CHF have been associated with the elevation of the Gal-3 levels. Furthermore, the efficacy of Valsartan treatment in preventing the need for hospitalization seems to be dependent upon the Gal-3 values, as suggested by Anand et al. [78]. It is yet unclear whether Gal-3 is influenced or not by other biomarkers, as suggested by the study performed by Miller et al., in which Gal-3 proved to be a predictor of cardiac adverse events in CHF patients only in conjunction with ST2 and not taken individually [79]. Given the extensive evidence available, Gal-3 has been proposed by The American College of Cardiology Foundation (ACCF) and the American Heart Association (AHA) as an integrated part of a biomarker panel that can assess the extent of cardiac fibrosis [80].

## 5. Gal-3 in Congenital Heart Disease

In healthy children, Gal-3 presents no variations in age and gender, as opposed to other biomarkers that are also altered in the setting of myocardial fibrosis, such as ST2, NT-proBNP and troponin [81,82]. These findings, provided by two pediatric studies that compared different age groups, are in line with the evidence-based theory of Schindler et al., which sustained the stability of Gal-3 over other cardiac biomarkers [83]. As a result, Gal-3 is considered to be a promising, age-independent biomarker in pediatric cardiovascular disorders that can identify early signs of cardiac disfunction. Table 1 overviews the role of Gal-3 in predicting the early onset of myocardial fibrosis, heart failure and a CHD outcome.

In CHD, the experimental data showed that Gal-3 is highly expressed in the setting of pulmonary artery hypertension (PAH), which represents one of the main causes of right ventricular failure and subsequent death. The pathomechanism involved is represented by the T-helper 2 (Th2) cell-induced release of inflammatory cytokines, such as interleukin(IL)-4 and IL-5 [84]. Thus, Gal-3 can prematurely identify the onset of right ventricle remodeling in patients with PAH and is positively associated with the right ventricular systolic and diastolic indices [85]. On the other hand, Gal-3 was not able to differentiate between adaptive and maladaptive RV remodeling in PAH patients within the study of Keranov et al. [86].

An association between Gal-3 and an right ventricle pressure overload was also revealed by a study performed on adult patients with CHD [87]. However, the prediction of the alteration of the cardiac function parameters and associated complications in children diagnosed with CHD, the main cause of CHF at pediatric ages, still remains a challenge. Gal-3 has emerged as a marker of progressive ventricular remodeling in children with CHD, which positively correlates with pulmonary artery pressure and the left atrial and ventricular diameters, according to Mohammed et al. [88]. Therefore, Gal-3 has also been analyzed in survivors of CHDs such as Tetralogy of Fallot (ToF) and has not been asserted as a potential biomarker candidate for long-term outcomes, as opposed to several other plasmatic biomarkers [89]. Kotby et al. identified an increase in the Gal-3 serum levels with chronic heart failure progression, quantified through Ross classification, regardless of ejection fraction preservation. Approximately one-third of their pediatric study group included patients with a CHD diagnosis, and the echocardiographic assessment yielded a negative correlation between Gal-3 and the systolic and diastolic function parameters [90]. The importance of Gal-3 in heart failure staging was also confirmed in a study conducted on children with CHD through a positive correlation between its serum levels and Ross classification [91]. However, while some authors (Saleh et al.) have claimed that Gal-3 might perform even better than Ross classification in the precocious identification of heart failure among children with CHD, exhibiting excellent sensitivity and specificity with a cut-off value of 10.4 ng/dL, some others (Cura et al.) proved in a case–control study that enrolled infants with ventricular septal defects (VSD) that Gal-3 increases independently from left ventricular dilation [92,93]. On the other hand, a study conducted on adults with CHD revealed that Gal-3 correlates with the global longitudinal strain (GLS) and NT-proBNP values but does not constitute a marker of major adverse cardiac events [94]. Another study (Geenen et al.) claimed that Gal-3 performs better than NT-proBNP or an echocardiographic strain analysis in predicting adverse outcome in adults with a systemic right ventricle and transposition of the great arteries (TGA) [95]. Therefore, given the conflicting results, the previously reported positive correlations between Gal-3 and the systolic and diastolic diameters, as well as end diastolic volumes, still require validation in larger cohorts [90,92].

A Gal-3 level increase is suggestive of the development of supraventricular and ventricular tachycardia in adults with CHD [94]. The presence of an arrhythmia brings about an additional enhancement of Gal-3 expression to the one normally found with a pre-existing disorder, as suggested by a study that comparatively assessed patients with CHD, rheumatic heart disease and rheumatic disease complicated by atrial fibrillation [96]. The correlation between Gal-3 and arrhythmia seems to be independent of a pre-existing underlying condition. A small-scale study discovered an increase in Gal-3 in adolescents with ventricular arrhythmias, as opposed to healthy counterparts. Moreover, the highest Gal-3 serum values were reported in patients with complex ventricular arrythmias. This finding was based on one study that revealed that this marker is dependent upon the severity of left ventricular dysfunction, as expressed through a moderate linear correlation between Gal-3 and the left ventricular diastolic diameter [97].

While taking into consideration the potential modulation of Gal-3 for the prevention of myocardial fibrosis, one study and two randomized controlled trials (RCTs) assessed the effect of an aldosterone antagonist agent, eplerenone, and a prostacyclin analogue, iloprost, on its circulating levels [98,99,100], while, in one RCT, eplerenone did not significantly influence the Gal-3 levels in adults with a childhood diagnosis of ToF or complete TGA. In another one, which enrolled adults with a history of TGA correction, a decrease in Gal-3 was found after one year of treatment [98,100]. In another study, iloprost inhalation did significantly lower the Gal-3 levels in patients with CHD and pulmonary artery hypertension. Thus, iloprost might influence myocardial fibrotic processes through Gal-3 modulation [99].

Gal-3 has also been proposed as a marker of unfavorable outcome in heritable cardiomyopathies. In the study of Hu et al., conducted on non-ischemic cardiomyopathy patients, Gal-3 correlated with major adverse cardiovascular events [101]. However, another study revealed a lack of correlation between Gal-3 and adverse outcomes in subjects with stable, dilated cardiomyopathy [102]. In vitro studies support the implications of Gal-3 in the ventricular remodeling implied by inherited cardiomyopathies. Thus, in a mouse model with dilated cardiomyopathy, deletion of the Gal-3 gene resulted in the attenuation of cardiac remodeling and dysfunction [103].

## 6. Gal-3 as a Predictor of Complications Associated with CHD Corrective Surgery

An integrated biomarker panel, consisting of ST2, Gal-3, glial fibrillary acidic protein (GFAP) and NT-proBNP, can constitute a relevant addition to clinical data in predicting the 30-day and one-year rehospitalization and mortality rates in children undergoing surgery for CHD [104,105,106]. Similar results were reported by Opotowsky et al., who found a positive relationship between the Gal-3 levels and the presence of Fontan circulation in adults, as well as a positive correlation with cardiovascular hospitalization and death [107]. Gal-3 has also been investigated as a potential predictor of post-operative ventricle remodeling in patients with CHD. DiLorenzo et al., who assessed multiple biomarkers of myocardial fibrosis for the detection of right ventricle remodeling after ToF surgical repair, amongst which was also Gal-3, concluded that only matrix metalloproteinase-1 (MMP-1), a marker of several common congenital cardiac and vascular anomalies, can really be taken into consideration [108,109]. In children undergoing surgery for isolated aortic coarctation with/without aortic hypoplasia, there was no significant linear relationship between the pre-operatory Gal-3 levels and post-operatory left ventricular mass index nor relative wall thickness, which were perceived as markers of left ventricular remodeling [110]. The prognostic role of Gal-3 in patients with functional univentricular hearts and Fontan circulation was also put into question by a study that did not include this biomarker amongst the ones related to major adverse events [111]. Table 2 provides details regarding the main outcomes of studies that investigated the role of Gal-3 in predicting myocardial fibrosis, as well as cardiovascular complications, in subjects who underwent CHD corrective surgery.

A decrease in the Gal-3 levels was reported immediately after the percutaneous closure of atrial septal defects in an adult cohort. However, the parameter returned to its baseline levels three months after the aforementioned procedure, irrespective of the cardiac remodeling processes [112]. On the other hand, Dudnyk et al. claimed that, even if children are completely asymptomatic in post-corrective surgery for CHD, their Gal-3 levels are still elevated and are associated with myocardial disfunction, depicted through modified tissue Doppler imaging measurements [113]. Another study conducted on adult patients confirmed that Gal-3 failed to reflect both left and right ventricle dysfunction in adult patients who had undergone surgical procedures for ToF repair at pediatric ages [114]. Furthermore, Gal-3 did not correlate with the hemodynamic parameters such as right atrial pressure or right ventricular end diastolic pressure in children undergoing transcatheter valve replacement for congenital pulmonary valve stenosis or insufficiency, unlike NT-proBNP and ST2 [115].

In children undergoing cardiopulmonary bypass during CHD corrective surgery, Gal-3 has also been studied in relation to acute kidney injury, occurring as a post-operative complication. Parsons et al. concluded that Gal-3 cannot be considered a reliable marker for acute kidney injury (AKI) in this clinical setting [116]. Contradictorily, Greenberg et al. described an independent association between the pre-operatory and first post-operatory Gal-3 levels and AKI in children undergoing cardiac surgical procedures [117].

## 7. Conclusions

From what is known so far, Gal-3 is a next-generation biomarker with predictive potential for cardiac dysfunction in right and left heart pathology in children and adults. This review provides comprehensive information regarding the potential utility of Gal-3 in predicting the early onset of heart failure signs and post-operatory complications of corrective surgery in patients with CHD. Its focus on the role of Gal-3 in CHD makes it peculiar among the previous literature reviews that have analyzed the same biomarker in cardiovascular diseases. The limitation of the present work is the heterogeneity and the lack of validation of data on large cohorts. A larger number of patients were enrolled in studies that assessed the role of Gal-3 in CHD post-corrective surgery scenarios. Further longitudinal research is required to establish how Gal-3 can relate to long-term outcomes and complications in both pediatric and adult CHD. The robustness of its predictor role depends on its placement in a clinical context and the integration of other parameters derived from an advanced cardiac imaging analysis (echocardiography and cardiac magnetic resonance).

## Figures and Tables

**Table 1:** Characteristics of clinical studies that assessed the role of Gal-3 in predicting myocardial disfunction and related complications associated with CHD.

Reference (Author, Year)	Type of Study	Population and Study Group Assignment	Main Outcome
Fenster et al., 2016 [85]	Case-control study	25 adult patients 15 patients with PAH in study group 10 patients in control group	Gal-3 levels were significantly higher in the study group when compared to the controls Significant association between Gal-3 and indices of RV systolic and diastolic function
Kowalik et al., 2020 [87]	Cross-sectional study	124 adult patients 63 patients with congenitally corrected TGA in study group I 41 patients with Eisenmenger syndrome in study group II 20 healthy controls	Increase in gal-3 levels within the study groups Gal-3 levels correlated with age, NYHA class, NT-proBNP and parameters of RV function only in patients with congenitally corrected TGA
Mohammed et al., 2014 [88]	Case-control study	90 pediatric patients 60 patients with left to right shunt CHD: 30 patients with manifestations of HF 30 patients without HF 30 healthy controls	Gal-3 levels were significantly higher in children with HF when compared with the ones of children without HF and the control group Significant association between Gal-3 levels and severity of HF, assessed with the help of Ross classification subclasses
Van den Bosch et al., 2022 [89]	Cross-sectional study	137 Fallot patients (adolescents and adults)	Gal-3 does not corelates with parameters of cardiac function and long-term outcome
Kotby et al., 2017 [90]	Cross-sectional study	90 pediatric patients 45 patients with CHF (15 patients with CHD): 22 patients with HFNEF (EF > 50%) 23 patients with HFREF (EF ≤ 50%) 45 healthy controls	Significant increase in Gal-3 levels within the study group No significant difference in serum Gal-3 levels between patients with HFNEF and those with HFREF
Elhewala et al., 2020 [91]	Case-control study	60 pediatric patients 40 patients with CHD: 20 patients with HF 20 patients without HF 20 healthy controls	Significant increase in Gal-3 levels within the study group Significantly higher Gal-3 levels were found in the study group in patients with HF as opposed to those without HF
Saleh et al., 2020 [92]	Case-control study	115 pediatric patients 75 patients with CHD 30 children with HFNEF 45 children with HFREF 40 healthy controls	Gal-3 might present an important predictive value for the early diagnosis of HF at a cut-off level of 10.4 ng/dl, with excellent sensitivity and specificity
Cura et al., 2022 [93]	Cross-sectional study	44 pediatric patients 22 infants with isolated VSD who had received HF treatment 22 healthy control infants	Significant increase in Gal-3 levels within the study group Gal-3 might be able to distinguish between individuals with the investigated pathology and controls at a cut-off level of 3.62 ng/mL, with good sensitivity and specificity
Frogoudaki et al., 2020 [94]	Cross-sectional study	58 adult patients with CHD: Group A—16 patients with no SVT episode Group B—27 patients with 1–2 episode of SVT or SV extrasystoles Group C—15 patients with multiple SVT episode or atrial fibrillation Group A’—15 patients with no VT episode Group B’—32 patients with ventricular extrasystoles or couplets Group C’—11 patients with triplets or non-sustained VT or more than 1000 extrasystoles	Gal-3 differed significantly between patient subgroups divided according to the presence and severity of SVT/VT Gal-3 did not correlate with major adverse cardiovascular events
Geenen et al., 2019 [95]	Prospective cohort study	86 adult patients with TGA and sRV: 65 patients with M-TGA 21 patients with ccTGA	Gal-3 level is a better predictor for death arrythmia than NT-proBNP and echocardiographic strain parameters
Xiao et al., 2020 [96]	Observational study	30 adult patients: 10 patients with CHD and normal sinus rhythm 10 patients with RHD and normal sinus rhythm 10 patients with RHD and AF	Expression levels of Gal-3 were increased in the human right atrial appendage tissue within the AF study group when compared to the other two study groups

Legend: AF—atrial fibrillation; ccTGA—congenitally corrected transposition of the great arteries; CHD—congenital heart disease; CHF—chronic heart failure; Gal-3—galectin 3; HF—heart failure; HFNEF—heart failure with normal ejection fraction; HFREF—heart failure with reduce ejection fraction; M-TGA—Mustard/Senning procedure applied for correction of transposition of the great arteries; NT-proBNP—N-terminal pro-brain natriuretic peptide; NYHA—New York Heart Association; PAH—pulmonary arterial hypertension; RCT—randomized control trials; RHD—rheumatic heart disease; RV—right ventricle; sRV—systemic right ventricle; SV—supraventricular; SVT—supraventricular tachycardia; TGA—transposition of the great arteries; VSD—ventricular septal defect; VT—ventricular tachycardia.

**Table 2:** Characteristics of clinical studies that assessed the role of Gal-3 in predicting myocardial disfunction and related complications in patients with CHD who underwent corrective surgery.

Reference (Author, Year)	Type of Study	Population and Study Group Assignment	Main Outcome
Parker et al., 2020 [98]	Longitudinal cohort study	145 pediatric patients with CHD and corrective surgery involving cardiopulmonary by-pass	An integrated biomarker panel, consisting of GFAP, NT-proBNP, ST2 and Gal-3 significantly correlated with an increased risk of readmission and mortality within the study cohort
Brown et al., 2019 [99]	Longitudinal cohort study	162 pediatric patients with CHD and corrective surgery	An integrated biomarker panel, consisting of GFAP, NT-proBNP, ST2 and Gal-3 significantly correlated with an increased risk of readmission and mortality 30 days after discharge within the study cohort
Parker et al., 2019 [104,105,106]	Longitudinal cohort study	162 pediatric patients with CHD and corrective surgery	An integrated biomarker panel, consisting of GFAP, NT-proBNP, ST2 and Gal-3 significantly correlated with an increased risk of readmission and mortality 30 days after discharge within the study cohort
Opotowsky et al., 2016 [101]	Longitudinal cohort study	70 adult patients who underwent Fontan procedure	High Gal-3 levels correlated with on elective cardiovascular hospitalization ad death
DiLorenzo et al., 2022 [102]	Longitudinal cohort study	60 pediatric patients who underwent corrective surgery for ToF	Gal-3 did not correlate with parameters of ventricular remodeling, depicted with the help of CMR
Frank et al., 2019 [110]	Longitudinal cohort study	27 pediatric patients who underwent aortic coarctation repair	Post-operatory Gal-3 levels did not vary significantly from the pre-operatory ones
van den Bosch et al., 2021 [111]	Longitudinal cohort study	133 pediatric patients who underwent the Fontan procedure	Gal-3 levels did not correlate with major adverse cardiovascular events
Geenen et al., 2020 [112]	Longitudinal cohort observational study	50 adult patients who underwent percutaneous ASD closure	Gal-3 returned to baseline levels three months after surgery
Dudnyk et al., 2019 [113]	Case-control study	224 pediatric patients: 184 patients with CHD and corrective surgery 40 healthy controls	Gal-3 values were significantly higher in the CHD cohort
Karali et al., 2021 [114]	Observational cohort study	35 adult patients with repaired ToF	No correlation found between Gal-3 and right ventricular myocardial fibrosis, quantified with the help of CMR Gal-3 levels correlated with moderate/severe pulmonary regurgitation
Zegelbone et al., 2020 [115]	Observational cohort study	16 adolescent and adult patients who underwent transcatheter pulmonary valve replacement	Gal-3 did not correlate with any atrial and ventricular function parameters, assessed through CMR, unlike NT-proBNP and ST2
Parsons et al., 2020 [116]	Observational cohort study	162 pediatric patients with CHD undergoing cardiopulmonary by-pass	Gal-3 levels did not differ significantly between children with post-operatory AKI and those without renal complications
Greenberg et al., 2021 [117]	Observational cohort study	395 pediatric patients with CHD undergoing cardiopulmonary by-pass: 194 children < 2 years of age 201 children ≥ 2 years of age	Gal-3 levels were independently associated with post-operative AKI but only in children ≥2 years of age

Legend: ASD—atrial septal defect; AKI—acute kidney injury; CHD—congenital heart disease; CMR—cardiac magnetic resonance; Gal-3—galectin 3; GFAP—glial fibrillary acidic protein; NT-proBNP—N-terminal pro-brain natriuretic peptide.

## Data Availability

Not applicable.
